# Prognostic influence of body mass index and body weight gain during adjuvant FOLFOX chemotherapy in Korean colorectal cancer patients

**DOI:** 10.1186/s12885-015-1704-0

**Published:** 2015-10-14

**Authors:** Dae-Won Lee, Sae-Won Han, Yongjun Cha, Kyung-Hun Lee, Tae-Yong Kim, Do-Youn Oh, Seock-Ah Im, Yung-Jue Bang, Ji Won Park, Seung-Bum Ryoo, Seung-Yong Jeong, Gyeong Hoon Kang, Kyu Joo Park, Tae-You Kim

**Affiliations:** 1 Department of Internal Medicine, Seoul National University Hospital, 101 Daehang-Ro, Jongno-Gu, Seoul, 110-744 South Korea; 2 Cancer Research Institute, Seoul National University, Seoul, South Korea; 3 Department of Surgery, Seoul National University Hospital, Seoul, South Korea; 4 Department of Pathology, Seoul National University Hospital, Seoul, South Korea; 5 Department of Molecular Medicine & Biopharmaceutical Sciences, Graduate School of Convergence Science and Technology, Seoul National University, Seoul, South Korea

**Keywords:** Body mass index, Obesity, FOLFOX, Chemotherapy, Colorectal cancer

## Abstract

**Background:**

Asian population has different body mass index (BMI) profile compared to Caucasian population. However, the effect of obesity and body weight gain in Asian colorectal cancer patients treated with adjuvant chemotherapy has not been studied thus far.

**Methods:**

We have analyzed the association between disease-free survival (DFS) and obesity/body weight change during treatment in Korean stage III or high-risk stage II colorectal cancer patients treated with adjuvant 5-fluorouracil/ leucovorin/oxaliplatin. BMI was classified according to WHO Asia-Pacific classification. Weight change was calculated by comparing body weights measured at the last chemotherapy cycle and before surgery.

**Results:**

Among a total of 522 patients, 35.7 % of patients were obese (BMI ≥ 25 kg/m^2^) and 29.1 % were overweight (BMI, 23–24.9 kg/m^2^) before surgery. 18.0 % of patients gained ≥ 5 kg and 26.1 % gained 2–4.9 kg during the adjuvant chemotherapy period. Baseline BMI or body weight change was not associated with DFS in the overall study population. However, body weight gain (≥5 kg) was associated with inferior DFS (adjusted hazard ratio 2.04, 95 % confidence interval 1.02–4.08, *p* = 0.043) in overweight and obese patients (BMI ≥ 23.0 kg/m^2^).

**Conclusion:**

In Korean colorectal cancer patients treated with adjuvant FOLFOX chemotherapy, body weight gain during the treatment period has a negative prognostic influence in overweight and obese patients.

## Background

Colorectal cancer is one of the leading causes of cancer incidence and death worldwide [1]. Although colorectal cancer mortality is declining in the developed countries, its incidence and mortality are increasing in other countries, including Korea, which are probably due to westernization of lifestyle [2, 3].

Body mass index (BMI) of ≥ 30.0 kg/m^2^ and 25.0–29.9 kg/m^2^ are proposed by the World Health Organization (WHO) for classification of obesity and overweight, respectively [4]. The prevalence of obesity using this definition is variable in different countries throughout the world [5]. According to the definition, the prevalence of obesity is higher than 20 % in many countries in Western Europe and North America, whereas it is less than 10 % in Asian countries including Korea and Japan [5]. Considerable efforts have been made to identify refined cut-offs of obesity for Asian countries in order to better reflect the health risk and provide appropriate action points [6, 7]. In Korea, BMI cut-off of 25 kg/m^2^ and 23.0–24.9 kg/m^2^ is used for the definition of obesity and overweight, respectively [8, 9].

While many epidemiology studies have shown that obesity increases the risk of developing colorectal cancer, there has been conflicting data on the prognostic impact of obesity in stage II and III colorectal cancer patients [10–15]. Most of the previous studies were conducted in Caucasian population and to our knowledge no study has investigated the effect of obesity in Asian colorectal patients treated with adjuvant chemotherapy thus far. Considering the increase in the prevalence of colorectal cancer and obesity in some Asian countries and the difference in BMI distribution between Asian and Caucasian, there is an urgent need for data from Asian patients. In addition, very limited data regarding body weight change after colorectal cancer treatment and its impact on treatment outcome is available [12]. Although body weight gain during the period of adjuvant chemotherapy is frequently observed in daily practice, its frequency and prognostic implication has not been studied in detail.

This study was undertaken to investigate how obesity and body weight change during chemotherapy influences prognosis in Korean colorectal cancer patients receiving adjuvant 5-fluorouracil, leucovorin, and oxaliplatin (FOLFOX) chemotherapy in order to provide preliminary answers to the above questions.

## Methods

### Patients and treatment

This retrospective analysis was performed with Korean patients who received curative surgery followed by adjuvant FOLFOX chemotherapy at Seoul National University Hospital (SNUH) (Seoul, Korea). Complete resection of the tumor followed by 12 cycles of FOLFOX chemotherapy is the current standard care in patients with stage III colon cancer [16]. Eligibility criteria for this study were: age over 18 years, adenocarcinoma histology, complete resection of the tumor with negative margins (from April 2005 to December 2011), stage III (any T, and N1 or N2M0) or high-risk stage II (T3 or T4N0M0), completion of at least 6 cycles of adjuvant FOLFOX, and adequate organ functions. High-risk stage II was defined if they had any of the following: T4 lesion, obstruction or perforation, lymphovascular invasion, perineural invasion, or poorly differentiated histology [17]. Patients with upper rectal cancer were included if the patient did not receive pre- or post-operative radiation. Only patients who received surgery and chemotherapy at SNUH were included. Patients were excluded if they had received previous chemotherapy or radiotherapy for colorectal cancer, if they had signet ring cell histology, distant metastasis or history of other malignancy within 5 years. Chemotherapy regimen was either FOLFOX-4 (from May 2005 to July 2009) or modified FOLFOX-6 (from July 2009 to December 2011) [18]. Adjuvant chemotherapy was planned for a total of 12 cycles and patients were assessed every 2 weeks during chemotherapy treatment, and then at least every 6 months for 5 years. The post chemotherapy period assessment included a medical history taking, physical examination, measurement of carcinoembryonic antigen level, chest computed tomography, and abdominal computed tomography. The diagnosis of recurrence was made on the basis of imaging and, if necessary, biopsy. From the electronic medical record system of SNUH, patients with diagnosis of colorectal cancer and prescription of oxaliplatin were retrieved. Patients fulfilling the inclusion and exclusion criteria by manual chart review were included in the study cohort. The study protocol was reviewed and approved by the institutional review board of SNUH, Seoul, Korea [H-1210-016-430]. As this study was retrospectively designed, informed consent was waived by the IRB. The study database was last updated in February 2014 (median follow-up duration 48 months). This study was carried out in accordance with the recommendations of the Declaration of Helsinki for biomedical research involving human subjects.

### Determination of BMI and body weight change

Trained nurses measured weight to the nearest 0.1 kg and height to the nearest 0.1 cm. BMI was calculated by dividing weight in kilograms by the square of height in meters. Patients were classified according to the BMI cut-offs proposed by WHO for Asian populations which is used in Korea: underweight, BMI < 18.5 kg/m^2^; normal range, 18.5–22.9; overweight, 23–24.9; obese I, 25–29.9; and obese II, ≥ 30 [6]. Body weight and height measured on the day of admission for colorectal cancer surgery was used for calculation of baseline body weight and BMI. For post chemotherapy body weight, we used the body weight measured at the last cycle of chemotherapy. Body weight change during treatment was calculated by comparing post chemotherapy body weight with baseline body weight.

### Statistical analysis

The primary objective of this study is to investigate the effects of BMI and body weight change during the periods of adjuvant FOLFOX chemotherapy on the treatment outcome (disease-free survival, DFS) of colorectal cancer patients. DFS was calculated from the date of operation to the first date of documented recurrence or the date of death from any cause. Data from patients who were free of recurrence were censored at the date of the last follow-up visit for DFS. Categorical variables were compared using chi-square test or Fisher’s exact test. Trend was analyzed using linear-by-linear association test. DFS was calculated using the Kaplan-Meier method and comparisons were made using the log-rank tests. Hazard ratios (HR) were calculated using the Cox proportional hazard model and baseline characteristics were adjusted by using backward stepwise model including covariates with a probability value ≤ 0.20 in the univariate analysis. Two-sided *p*-values of less than 0.05 were considered statistically significant. Statistical analysis was performed with SPSS software for Windows, version 18.0 (SPSS, Chicago, IL, USA).

## Results

### Patient characteristics and BMI

Baseline characteristics of 522 patients included in the present study are summarized in Table 1. The mean baseline BMI of our cohort was 24.0 kg/m^2^ (male: 24.1 kg/m^2^, female: 23.9 kg/m^2^). Based on the cut-offs proposed by WHO for Asians [6], 10 patients (1.9 %) were underweight (BMI < 18.5 kg/m^2^), 174 patients (33.3 %) were normal weight (18.5–22.9 kg/m^2^), 152 patients (29.1 %) were overweight (23–24.9 kg/m^2^), 171 patients (32.8 %) were obese I (25–29.9 kg/m^2^) and 15 patients (2.9 %) were obese II (≥ 30 kg/m^2^). Due to the limited numbers of patients with underweight or obese II, we classified patients into 3 groups in further statistical analysis: normal or underweight, ≤ 22.9 kg/m^2^; overweight, 23–24.9 kg/m^2^; and obese, BMI ≥ 25 kg/m^2^.

**Table 1:** Baseline characteristics

		Body Mass Index			
	Total	Normal or underweight	Overweight	Obese	*p-*value*
		≤ 22.9 kg/m^2^	23–24.9 kg/m^2^	≥ 25 kg/m^2^	
	N (%)	N (%)	N (%)	N (%)	
Total	522 (100)	184 (35.2)	152 (29.1)	186 (35.6)	
Age					0.028
< 65 years	366 (70.1)	138 (75.0)	108 (71.1)	120 (64.5)	0.42
≥ 65 years	156 (29.9)	46 (25.0)	44 (28.9)	66 (35.5)	0.028
Sex					0.094
Male	312 (59.8)	99 (53.8)	97 (63.8)	116 (62.4)	0.064
Female	210 (40.2)	85 (46.2)	55 (36.2)	70 (37.6)	0.095
Location					0.56
Proximal	181 (34.7)	63 (34.2)	49 (32.2)	69 (37.1)	0.70
Distal	341 (65.3)	121 (65.8)	103 (67.8)	117 (62.9)	0.57
T stage					0.17
T1 - 3	446 (85.4)	149 (81.0)	137 (90.1)	160 (86.0)	0.019
T4	76 (14.6)	35 (19.0)	15 (9.9)	26 (14.0)	0.19
N stage					0.96
N0 - 1	380 (72.8)	133 (72.3)	113 (74.3)	134 (72.0)	0.67
N2	142 (27.2)	51 (27.7)	39 (25.7)	52 (28.0)	0.96
Tumor stage					0.073
II, high-risk	78 (14.9)	37 (20.1)	16 (10.5)	25 (13.4)	0.016
III	444 (85.1)	147 (79.9)	136 (89.5)	161 (86.6)	0.086
Histology					0.15
MAC	27 (5.2)	12 (6.5)	9 (5.9)	6 (3.2)	0.82
Non-MAC	495 (94.8)	172 (93.5)	143 (94.1)	180 (96.8)	0.14
Microsatellite status (*N* = 517)					0.062
MSS/MSI-L	480 (92.8)	163 (90.1)	142 (93.4)	175 (95.1)	0.27
MSI-H	37 (7.2)	18 (9.9)	10 (6.6)	9 (4.9)	0.065

Abbreviations: *MAC* mucinous adenocarcinoma, *MSS* microsatellite stable, *MSI-L* microsatellite instability-low, *MSI-H* microsatellite instability-high

*Upper row: linear-by-linear association test, middle row: *Χ*^*2*^ test of normal or underweight *vs.* overweight, lower row: *Χ*^2^ test of normal or underweight *vs.* obese

Obese patients had higher proportion of older (≥ 65 years, *p* = 0.028) and male (*p* = 0.095) population compared with normal or underweight patients. The frequency of stage III disease (*p* = 0.086) and MSI-high tumors (*p* = 0.065) tended to be higher in obese patients. Tumor location (proximal *vs.* distal) was similar among the BMI groups (Table 1). According to the inclusion criteria, all patients received at least 6 cycles of chemotherapy and 463 patients (88.7 %) completed planned 12 cycles of chemotherapy. There was no difference in chemotherapy completion rate according to BMI status (*p* = 0.49).

### Body weight change during chemotherapy

In the classification of body weight change, we adopted the cut-off value of weight gain or loss of 2 kg and 5 kg, which has been utilized in a previous study [12]. A total of 44.1 % of patients gained body weight during adjuvant chemotherapy (Table 2). Ninety-four patients (18.0 %) gained 5 or more kilograms and 136 patients (26.1 %) gained 2–4.9 kg. In contrast, 18.4 % of patients lost body weight. Eighty patients (15.3 %) lost 2.1–5 kg and 16 patients (3.1 %) lost more than 5 kg.

**Table 2:** Body weight change and baseline characteristics

	Total	> 5 kg loss	2.1–5 kg loss	± 2 kg	2–4.9 kg gain	≥ 5 kg gain	*p*-value*
		N (%)	N (%)	N (%)	N (%)	N (%)	
Total	522	16 (3.1)	80 (15.3)	196 (37.5)	136 (26.1)	94 (18.0)	
Baseline BMI^a^							
Normal or underweight	184	2 (1.1)	16 (8.7)	63 (34.2)	46 (25.0)	57 (31.0)	< 0.001
Overweight	152	3 (2.0)	23 (15.1)	58 (38.2)	45 (29.6)	23 (15.1)	< 0.001
Obese	186	11 (5.9)	41 (22.0)	75 (40.3)	45 (24.2)	14 (7.5)	
Age							
< 65 years	366	11 (3.0)	52 (14.2)	128 (35.0)	97 (26.5)	78 (21.3)	0.006
≥ 65 years	156	5 (3.2)	28 (17.9)	68 (43.6)	39 (25.0)	16 (10.3)	0.003
Sex							
Male	312	11 (3.5)	57 (18.3)	115 (36.9)	71 (22.8)	58 (18.6)	0.11
Female	210	5 (2.4)	23 (11.0)	81 (38.6)	65 (31.0)	36 (17.1)	0.67
Location							
Proximal	181	3 (1.7)	30 (16.6)	66 (36.5)	45 (24.9)	37 (20.4)	0.40
Distal	341	13 (3.8)	50 (14.7)	130 (38.1)	91 (26.7)	57 (16.7)	0.29
T stage							
T1 - 3	446	16 (3.6)	67 (15.0)	175 (39.2)	112 (25.1)	76 (17.0)	0.056
T4	76	0 (0.0)	13 (17.1)	21 (27.6)	24 (31.6)	18 (23.7)	0.16
N stage							
N0 - 1	380	10 (2.9)	61 (16.1)	142 (37.4)	105 (27.6)	62 (16.3)	0.55
N2	142	6 (4.2)	19 (13.4)	54 (38.0)	31 (21.8)	32 (22.5)	0.10
Tumor stage							
II, high-risk	78	2 (2.6)	9 (11.5)	24 (30.8)	29 (37.2)	14 (17.9)	0.15
III	444	14 (3.2)	71 (16.0)	172 (38.7)	107 (24.1)	80 (18.0)	1.00
Histology							
MAC	27	1 (3.7)	5 (18.5)	6 (22.2)	5 (18.5)	10 (37.0)	0.180
Non-MAC	495	15 (3.0)	75 (15.2)	190 (38.4)	131 (26.5)	84 (17.0)	0.017^**^
Microsatellite status (*N* = 517)							
MSS + MSI-L	480	14 (2.9)	73 (15.2)	186 (38.8)	124 (25.8)	83 (17.3)	0.33
MSI-H	37	1 (2.7)	7 (18.9)	9 (24.3)	10 (27.0)	10 (27.0)	0.14

Abbreviations: *MAC* mucinous adenocarcinoma, *MSS* microsatellite stable, *MSI-L* microsatellite instability-low, *MSI-H* microsatellite instability-high

*Upper row: linear-by-linear association test, lower row: *Χ*^*2*^ test of ≥ 5 kg gain group *vs.* the others

^**^Fisher’s exact test of ≥ 5 kg gain group *vs.* the others

^a^Normal or underweight, ≤ 22.9 kg/m^2^; overweight, 23–24.9 kg/m^2^; and obese, BMI ≥ 25 kg/m^2^

Patients who had lower baseline BMI (*p* < 0.001) and younger (< 65 years, *p* = 0.003) were more likely to gain body weight (≥ 5 kg gain) during the adjuvant chemotherapy period. Location of the tumor did not influence body weight gain (≥ 5 kg gain) during the adjuvant chemotherapy period (proximal *vs.* distal = 20.4 % *vs.* 16.7 %, *p* = 0.29).

### Impact of baseline BMI and body weight change on DFS

**Fig. 1:**
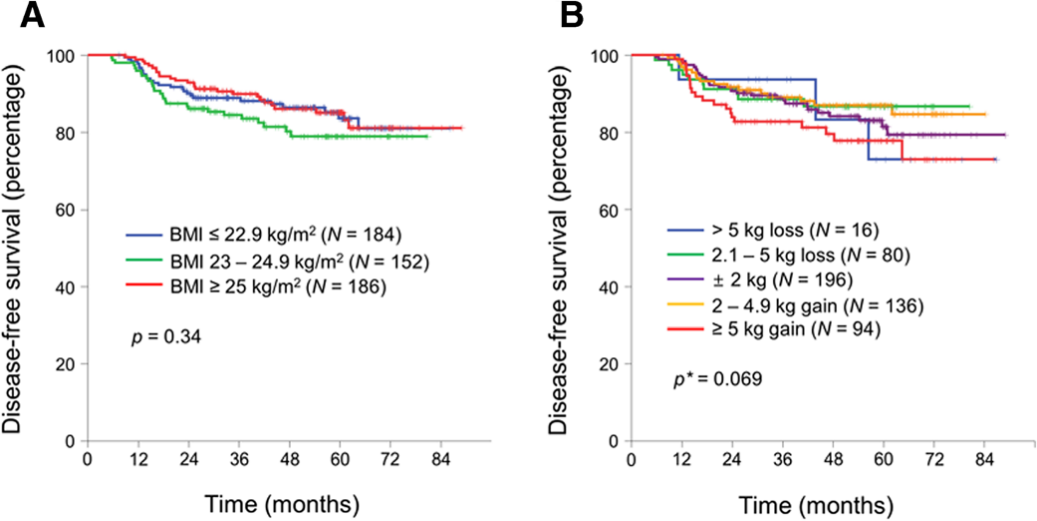
Kaplan-Meier curves of disease-free survival according to baseline BMI (**a**) and body weight change (**b**). *p-value of weight gain ≥ 5 kg *vs.* others

There was no significant difference in DFS according to the baseline BMI groups (Fig. 1a). Three-year DFS were 90.0 % in obese patients, 84.5 % in overweight, and 89.0 % in normal or underweight (*p* = 0.34). In addition, pattern of recurrence (local recurrence *vs.* distant metastasis) was similar between each baseline BMI groups (data not shown). We have also analyzed whether BMI status has a prognostic role in subgroups of patients. However, baseline BMI status was not associated with DFS in any clinico-pathological subgroups including the sex (data not shown).

We next evaluated the influence of body weight change on DFS. Patients with body weight gain of ≥ 5 kg (3-year DFS 82.8 %) showed tendency towards worse prognosis compared to the other patients (3-year DFS 89.2 %; *p* = 0.069) (Fig. 1b).

**Fig. 2:**
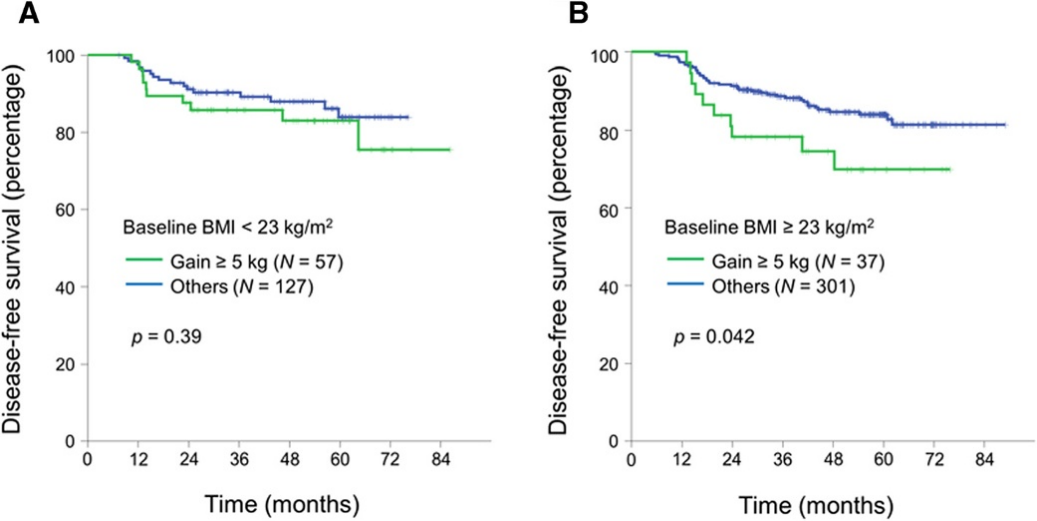
Kaplan-Meier curves of disease-free survival according to body weight change stratified by baseline BMI. **a** Baseline BMI < 23 kg/m^2^, **b** Baseline BMI ≥ 23 kg/m^2^

We hypothesized that body weight gain may have different patho-physiologic effect in overweight or obese patients compared with normal or underweight patients. Therefore, we analyzed the association between body weight gain (≥ 5 kg) and DFS stratified by baseline BMI. In the normal or underweight patient population (BMI ≤ 22.9 kg/m^2^), there was no significant difference in DFS according to body weight gain (≥ 5 kg) (Fig. 2a). In contrast, body weight gain (≥ 5 kg) was associated with significantly worse DFS in overweight or obese patient population (BMI ≥ 23.0 kg/m^2^) (Fig. 2b, Table 3). In the multivariate analysis, the poor prognosis associated with weight gain (≥ 5 kg) in the overweight or obese patient population was independent of other clinico-pathologic prognostic factors (adjusted hazard ratio 2.04, 95 % confidence interval 1.02–4.08) (Table 4).

**Table 3:** Univariate analysis of disease free survival among patients with baseline BMI ≥ 23 kg/m^2^ (*N* = 338)

		Unadjusted HR (95 % CI)	*p*-value
Sex	Male	1.46 (0.82–2.63)	0.20
	Female	1	
Age	≥ 65 years	1.09 (0.62–1.90)	0.77
	< 65 years	1	
Body weight change	Gain ≥ 5 kg	2.01 (1.01–3.99)	0.047
	Others	1	
Location	Proximal	0.85 (0.48–1.50)	0.57
	Distal	1	
Stage	III	1.32 (0.53–3.31)	0.55
	II	1	
T stage	T 4	2.53 (1.36–4.72)	0.004
	T 1–3	1	
N stage	N 2	2.96 (1.74–5.05)	<0.001
	N 0–1	1	
Angiolymphatic invasion	Present	3.87 (2.11–7.12)	<0.001
	Absent	1	
Venous invasion	Present	2.50 (1.26–4.98)	0.009
	Absent	1	
Perineural invasion	Present	3.53 (2.05–6.07)	<0.001
	Absent	1	
Histology	MAC	1.35 (0.42–4.34)	0.61
	Non-MAC	1	
Microsatellite status	MSI-H	0.70 (0.17–2.87)	0.62
	MSS/MSI-L	1	

Abbreviations: *HR* hazard ratio, *CI* confidence interval, *MAC* mucinous adenocarcinoma, *MSS* microsatellite stable, *MSI-L* microsatellite instability-low, *MSI-H* microsatellite instability-high

**Table 4:** Multivariate analysis of disease free survival among patients with baseline BMI ≥ 23 kg/m^2^ (*N* = 338)

		Adjusted HR (95 % CI)	*p*-value
Body weight change	Gain ≥ 5 kg	2.04 (1.02–4.08)	0.043
	Others	1	
Angiolymphatic invasion	Present	2.61 (1.37–4.94)	0.003
	Absent	1	
Perineural invasion	Present	2.51 (1.43–4.38)	0.001
	Absent	1	
T stage	T 4	1.99 (1.05–3.76)	0.035
	T 1–3	1	
N stage	N 2	2.34 (1.35–4.06)	0.002
	N 0–1	1	

Abbreviations: *HR* hazard ratio, *CI* confidence interval

## Discussion

In the present study, we have investigated the impact of obesity and body weight change during chemotherapy on the treatment outcome of Korean colorectal cancer patients receiving adjuvant FOLFOX chemotherapy. While obesity and colorectal cancer has become one of the major health problems in Asian countries including Korea, there has been paucity of data regarding obesity among Asian colorectal cancer patients [19, 20]. To our knowledge this is the first Asian study to evaluate the prognostic role of body weight change during adjuvant chemotherapy treatment. We observed that baseline BMI is not associated with DFS, but body weight gain (≥ 5 kg) during the adjuvant chemotherapy has deleterious effect in overweight or obese patients (BMI ≥ 23.0 kg/m^2^).

Although there has been some controversy in the prognostic role of obesity in stage II or III colorectal cancer patients, most data suggest negative prognostic role of obesity. From the result of Adjuvant Colon Cancer Endpoints (ACCENT) database, which is a pooled resource of over 20,000 colon cancer patients in adjuvant chemotherapy trials, obesity was associated with inferior outcome [15]. The adverse prognostic effect was only observed in male patients, but there are inconsistent data regarding gender-related prognostic difference of obesity [10, 14, 15].

However, these studies were all conducted in Western countries where BMI distribution is different form Asian population. In our study, 35.6 % of patients had BMI ≥ 25.0 kg/m^2^ and only 2.9 % of patients had BMI ≥ 30 kg/m^2^, which are similar to the incidence of Taiwanese and Japanese colorectal cancer patients (4.2 and 1.5 %, respectively) [19, 20]. In contrast, 53.6 % of patients have BMI ≥ 25.0 kg/m^2^ and 17.6 % have BMI ≥ 30 kg/m^2^ in the ACCENT study population [15]. Because of such a difference in BMI distribution between Asian and Caucasian population, we could not directly apply the findings from Western data to Asian colorectal cancer patients.

There are many studies evaluating the prognostic role of weight gain after cancer diagnosis in breast cancer patients [21–23]. Weight gain after breast cancer diagnosis was associated with higher rates of recurrence and mortality [21]. However, only one study has evaluated the prognostic role of weight change in stage III colon cancer patients [12]. In contrast to breast cancer, there was no prognostic role of weight gain in stage III colon cancer patients in the Cancer and Leukemia Group B 89803 study which examined the addition of irinotecan to adjuvant 5-fluorouracil and leucovorin [12]. In the present study, while there was no prognostic role of body weight change in the entire cohort, weight gain was a negative prognostic factor in overweight or obese patients. The discordant results may be attributable to differences in ethnicity, BMI distribution, chemotherapy regimen, and timing of body weight measurements. Meyerhardt and colleagues compared body weights measured at 4 months (in the middle of adjuvant chemotherapy) and 14 months after surgery (6 months after completion of chemotherapy) [12]. As we measured body weight change during the adjuvant chemotherapy period (before surgery and at the last cycle of chemotherapy), body weight gain in the present study may reflect the direct influence of weight gain on chemotherapy sensitivity. Obesity leads to decreased level of circulating adiponectin and increased level of insulin-like growth factor 1 and leptin, which contribute to an increased risk of colorectal cancer [24]. Moreover, there are data that tumor expression of leptin is associated with chemotherapy resistance [25]. Therefore, it is tempting to speculate that body weight gain during the adjuvant chemotherapy period may have altered adipokine levels, which in turn contributed to chemotherapy resistance. Dynamic changes in adipokine levels might be more deleterious than the baseline BMI and the deleterious effect is potentiated in the higher BMI patients. However, because of limited number of patients, we could not evaluate effect size between weight gain and DFS. Future large prospective study is needed to confirm our result.

Adding expensive targeted agents such as cetuximab or bevacizumab to adjuvant chemotherapy have failed to decrease recurrence in stage III colon cancer patients [26–29]. However, physical activity appears to reduce the risk of cancer recurrence and mortality in stage III colon cancer patients enrolled in a randomized adjuvant chemotherapy trial [30]. In patients receiving adjuvant chemotherapy, life style modification may be a cost effective approach to improve treatment outcome. Future prospective study is needed to confirm whether life style interventions can improve the outcome. In the meantime, patients may be advised to maintain their body weight during adjuvant chemotherapy period.

There are several limitations in our study. This study was retrospectively designed that we lacked other important data of body habitus such as weight circumference, fat distribution and lifestyle factors including diet, physical activity and smoking that may have interaction with BMI and weight change. Moreover, we do not have body weight data during follow-up period after completion of chemotherapy. Therefore, it is essential to establish an Asian prospective colorectal cancer cohort to overcome these limitations and comprehensively study the impact of obesity and lifestyle factors on prognosis of Asian patients. Another limitation is the relatively small sample size that we could not perform more detailed analyses including sub-analysis according to patient sex. However, the patient cohort was homogenous that all patients received surgery at a high-volume center and received same adjuvant chemotherapy regimen. Lastly, we could not evaluate overall survival due to limited number of death events during the relatively short duration of follow-up, albeit the 3-year DFS has been demonstrated to have a good correlation with 5-year overall survival in colon cancer [31].

## Conclusions

Baseline BMI was not associated with prognosis in Korean colorectal cancer patients treated with adjuvant FOLFOX chemotherapy. However, body weight gain during adjuvant chemotherapy had a negative prognostic impact in overweight or obese patients. Until future prospective study confirms our finding, overweight or obese patients may be advised to maintain their body weight during the adjuvant chemotherapy period.

## Abbreviations

ACCENT: Adjuvant Colon Cancer Endpoints
BMI: Body mass index
DFS: Disease-free survival
FOLFOX: 5-fluorouracil, leucovorin, and oxaliplatin
HR: Hazard ratio
CI: Condidence interval
MAC: Mucinous adenocarcinoma
MSS: Microsatellites stable
MSI-L: Microsatellite instability-low
MSI-H: Microsatellite instability-high
SNUH: Seoul National University Hospital
WHO: World Health Organization.

## References

[1] Jemal A, Bray F, Center MM, Ferlay J, Ward E, Forman D (2011). Global cancer statistics. CA Cancer J Clin.

[2] Center MM, Jemal A, Smith RA, Ward E (2009). Worldwide variations in colorectal cancer. CA Cancer J Clin.

[3] Jung KW, Won YJ, Kong HJ, Oh CM, Lee DH, Lee JS (2014). Cancer statistics in Korea: incidence, mortality, survival, and prevalence in 2011. Cancer Res Treat.

[4] WHO (2000). Obesity: preventing and managing the global epidemic [report of a WHO Consultation on Obesity].

[5] Ng M, Fleming T, Robinson M, Thomson B, Graetz N, Margono C, Mullany EC, Biryukov S, Abbafati C, Abera SF (2014). Global, regional, and national prevalence of overweight and obesity in children and adults during 1980-2013: a systematic analysis for the Global Burden of Disease Study 2013. Lancet.

[6] WHO (2000). The Asia-Pacific perspective: redefining obesity and its treatment.

[7] Consultation WE (2004). Appropriate body-mass index for Asian populations and its implications for policy and intervention strategies. Lancet.

[8] Kim CS, Ko SH, Kwon HS, Kim NH, Kim JH, Lim S, Choi SH, Song KH, Won JC, Kim DJ (2014). Prevalence, awareness, and management of obesity in Korea: data from the Korea national health and nutrition examination survey (1998-2011). Diabetes Metab J.

[9] Oh SW, Shin SA, Yun YH, Yoo T, Huh BY (2004). Cut-off point of BMI and obesity-related comorbidities and mortality in middle-aged Koreans. Obes Res.

[10] Meyerhardt JA, Catalano PJ, Haller DG, Mayer RJ, Benson AB, Macdonald JS, Fuchs CS (2003). Influence of body mass index on outcomes and treatment-related toxicity in patients with colon carcinoma. Cancer.

[11] Meyerhardt JA, Tepper JE, Niedzwiecki D, Hollis DR, McCollum AD, Brady D, O’Connell MJ, Mayer RJ, Cummings B, Willett C (2004). Impact of body mass index on outcomes and treatment-related toxicity in patients with stage II and III rectal cancer: findings from Intergroup Trial 0114. J Clin Oncol.

[12] Meyerhardt JA, Niedzwiecki D, Hollis D, Saltz LB, Mayer RJ, Nelson H, Whittom R, Hantel A, Thomas J, Fuchs CS (2008). Impact of body mass index and weight change after treatment on cancer recurrence and survival in patients with stage III colon cancer: findings from Cancer and Leukemia Group B 89803. J Clin Oncol.

[13] Dignam JJ, Polite BN, Yothers G, Raich P, Colangelo L, O’Connell MJ, Wolmark N (2006). Body mass index and outcomes in patients who receive adjuvant chemotherapy for colon cancer. J Natl Cancer Inst.

[14] Sinicrope FA, Foster NR, Sargent DJ, O’Connell MJ, Rankin C (2010). Obesity is an independent prognostic variable in colon cancer survivors. Clin Cancer Res.

[15] Sinicrope FA, Foster NR, Yothers G, Benson A, Seitz JF, Labianca R, Goldberg RM, Degramont A, O’Connell MJ, Sargent DJ (2013). Body mass index at diagnosis and survival among colon cancer patients enrolled in clinical trials of adjuvant chemotherapy. Cancer.

[16] Andre T, Boni C, Navarro M, Tabernero J, Hickish T, Topham C, Bonetti A, Clingan P, Bridgewater J, Rivera F (2009). Improved overall survival with oxaliplatin, fluorouracil, and leucovorin as adjuvant treatment in stage II or III colon cancer in the MOSAIC trial. J Clin Oncol.

[17] Schmoll HJ, Van Cutsem E, Stein A, Valentini V, Glimelius B, Haustermans K, Nordlinger B, van de Velde CJ, Balmana J, Regula J (2012). ESMO Consensus Guidelines for management of patients with colon and rectal cancer. a personalized approach to clinical decision making. Ann Oncol.

[18] Lee DW, Han SW, Lee HJ, Rhee YY, Bae JM, Cho NY, Lee KH, Kim TY, Oh DY, Im SA (2013). Prognostic implication of mucinous histology in colorectal cancer patients treated with adjuvant FOLFOX chemotherapy. Br J Cancer.

[19] Chin CC, Kuo YH, Yeh CY, Chen JS, Tang R, Changchien CR, Wang JY, Huang WS (2012). Role of body mass index in colon cancer patients in Taiwan. World J Gastroenterol.

[20] Yamamoto N, Fujii S, Sato T, Oshima T, Rino Y, Kunisaki C, Masuda M, Imada T (2012). Impact of body mass index and visceral adiposity on outcomes in colorectal cancer. Asia Pac J Clin Oncol.

[21] Kroenke CH, Chen WY, Rosner B, Holmes MD (2005). Weight, weight gain, and survival after breast cancer diagnosis. J Clin Oncol.

[22] Chen X, Lu W, Zheng W, Gu K, Chen Z, Zheng Y, Shu XO (2010). Obesity and weight change in relation to breast cancer survival. Breast Cancer Res Treat.

[23] Thivat E, Therondel S, Lapirot O, Abrial C, Gimbergues P, Gadea E, Planchat E, Kwiatkowski F, Mouret-Reynier MA, Chollet P (2010). Weight change during chemotherapy changes the prognosis in non metastatic breast cancer for the worse. BMC Cancer.

[24] Bardou M, Barkun AN, Martel M (2013). Obesity and colorectal cancer. Gut.

[25] Bain GH, Collie-Duguid E, Murray GI, Gilbert FJ, Denison A, McKiddie F, Ahearn T, Fleming I, Leeds J, Phull P (2014). Tumour expression of leptin is associated with chemotherapy resistance and therapy-independent prognosis in gastro-oesophageal adenocarcinomas. Br J Cancer.

[26] Taieb J, Tabernero J, Mini E, Subtil F, Folprecht G, Van Laethem JL, Thaler J, Bridgewater J, Petersen LN, Blons H (2014). Oxaliplatin, fluorouracil, and leucovorin with or without cetuximab in patients with resected stage III colon cancer (PETACC-8): an open-label, randomised phase 3 trial. Lancet Oncol.

[27] Allegra CJ, Yothers G, O’Connell MJ, Sharif S, Petrelli NJ, Colangelo LH, Atkins JN, Seay TE, Fehrenbacher L, Goldberg RM (2011). Phase III trial assessing bevacizumab in stages II and III carcinoma of the colon: results of NSABP protocol C-08. J Clin Oncol.

[28] de Gramont A, Van Cutsem E, Schmoll HJ, Tabernero J, Clarke S, Moore MJ, Cunningham D, Cartwright TH, Hecht JR, Rivera F (2012). Bevacizumab plus oxaliplatin-based chemotherapy as adjuvant treatment for colon cancer (AVANT): a phase 3 randomised controlled trial. Lancet Oncol.

[29] Alberts SR, Sargent DJ, Nair S, Mahoney MR, Mooney M, Thibodeau SN, Smyrk TC, Sinicrope FA, Chan E, Gill S (2012). Effect of oxaliplatin, fluorouracil, and leucovorin with or without cetuximab on survival among patients with resected stage III colon cancer: a randomized trial. JAMA.

[30] Meyerhardt JA, Heseltine D, Niedzwiecki D, Hollis D, Saltz LB, Mayer RJ, Thomas J, Nelson H, Whittom R, Hantel A (2006). Impact of physical activity on cancer recurrence and survival in patients with stage III colon cancer: findings from CALGB 89803. J Clin Oncol.

[31] Sargent DJ, Wieand HS, Haller DG, Gray R, Benedetti JK, Buyse M, Labianca R, Seitz JF, O’Callaghan CJ, Francini G (2005). Disease-free survival versus overall survival as a primary end point for adjuvant colon cancer studies: individual patient data from 20,898 patients on 18 randomized trials. J Clinical Oncol.

